# Engineered RBCs Encapsulating Antigen Induce Multi-Modal Antigen-Specific Tolerance and Protect Against Type 1 Diabetes

**DOI:** 10.3389/fimmu.2022.869669

**Published:** 2022-04-04

**Authors:** Colin J. Raposo, Judith D. Cserny, Gloria Serena, Jonathan N. Chow, Patricia Cho, Hanyang Liu, David Kotler, Armon Sharei, Howard Bernstein, Shinu John

**Affiliations:** SQZ Biotechnologies, Watertown, MA, United States

**Keywords:** red blood cells, regulatory T cells, tolerance, cell therapy, immunotherapy, autoimmunity, type 1 diabetes, antigens specific tolerance

## Abstract

Antigen-specific therapies that suppress autoreactive T cells without inducing systemic immunosuppression are a much-needed treatment for autoimmune diseases, yet effective strategies remain elusive. We describe a microfluidic Cell Squeeze^®^ technology to engineer red blood cells (RBCs) encapsulating antigens to generate tolerizing antigen carriers (TACs). TACs exploit the natural route of RBC clearance enabling tolerogenic presentation of antigens. TAC treatment led to antigen-specific T cell tolerance towards exogenous and autoantigens in immunization and adoptive transfer mouse models of type 1 diabetes (T1D), respectively. Notably, in several accelerated models of T1D, TACs prevented hyperglycemia by blunting effector functions of pathogenic T cells, particularly in the pancreas. Mechanistically, TACs led to impaired trafficking of diabetogenic T cells to the pancreas, induced deletion of autoreactive CD8 T cells and expanded antigen specific Tregs that exerted bystander suppression. Our results highlight TACs as a novel approach for reinstating immune tolerance in CD4 and CD8 mediated autoimmune diseases.

## Introduction

Autoreactive T cells play a central role in the pathogenesis and perpetuation of several autoimmune diseases (1). Current therapies for autoimmune diseases are focused on broad immunosuppression, which can limit efficacy and increase the risk of infection and cancer (2). Antigen-specific immune therap'ies (ASIT) that precisely target autoreactive T cells while sparing the normal immune cells would represent the therapeutic “holy grail” for the treatment of autoimmune diseases. ASITs aim to reset the immune system by inducing one or more mechanisms of peripheral tolerance. Tolerance to self-antigens in the periphery is maintained by anergy, deletion or suppression by regulatory T (Treg) cells (3, 4). Several ASIT approaches employing autoantigens in different formats including native and altered peptides, native or modified proteins, nucleic acid vaccines, peptide-MHC complexes, nanomedicines, antigen-conjugated apoptotic cells, and engineered dendritic Cells (DC) or Treg cells have either undergone clinical testing with limited success or are being currently evaluated (2, 5–11).

Among the various ASIT approaches, the use of apoptotic cells to deliver autoantigen has garnered traction due to its natural clearance in a tolerogenic fashion. Removal of apoptotic cells is carried out by macrophages and other professional phagocytes in a non-inflammatory manner, a process known as efferocytosis (12). Each day, billions of red blood cells (RBC) undergo a unique form of apoptosis known as eryptosis and are similarly phagocytosed by professional antigen presenting cells (APC) primarily in spleen, bone marrow and liver (13). Efferocytosis results in secretion of anti-inflammatory cytokines and antigens associated with apoptotic cells or eryptotic RBCs are processed and presented in a tolerogenic manner (14, 15).

Several strategies exploiting the clearance of apoptotic cells have been explored for the induction of peripheral tolerance and have demonstrated varying degrees of efficacy in several preclinical models (16–24). However, all these approaches are costly and have complex manufacturing, requiring different methods or modifications to couple or load antigens to apoptotic leukocytes or RBCs (16, 25, 26). The cell squeeze platform offers an attractive alternative through its high throughput microfluidic approach that obviates the need for complex antigen delivery techniques. The microfluidic device has parallel channels consisting of constrictions that have a dimension generally smaller than the diameter of the cell. Passage of cells through the narrow constriction causes temporary disruption of the cell membrane enabling passive diffusion of the target material from surrounding buffer into the cell cytosol (27). The cell membrane is thereafter resealed entrapping the target material (28). The microfluidic squeeze method has been used for delivery of a variety of materials such as proteins, mRNA and nanoparticles into different cell types (27, 29).

Here, we describe the use of the microfluidic cell squeeze platform to engineer RBCs encapsulating antigens to generate tolerizing antigen carriers (TACs), which resemble eryptotic RBCs in certain aspects, enabling their clearance by a natural route. TAC treatment in immunization and accelerated models of T1D led to induction of antigen-specific tolerance in CD4 and CD8 T cells. TAC induced tolerance was mediated by antigen-specific deletion of T cells or expansion of antigen-specific Tregs that exhibited potent bystander suppression. These results demonstrate the versatility of the TAC platform in inducing antigen-specific tolerance for T cell mediated autoimmune diseases.

## Materials and Methods

### Mice

All studies were carried out according to protocols established by the American Association for Laboratory Animal Science (IACUC) committee at SQZ Biotechnologies Company. Mice were procured from The Jackson Laboratory (Bar Harbor, ME) and maintained in a specific pathogen-free facility at SQZ Biotechnologies Company. Female C57BL/6J (JAX 664) mice aged 8-16 weeks were utilized for assessment of TAC clearance and biodistribution, as well as in the Ovalbumin (OVA) challenge model. Splenectomy and sham surgeries were performed by The Jackson Laboratory, at 7 weeks of age. For accelerated adoptive transfer models of Type 1 Diabetes (T1D), lymphocytes were isolated from either female donor NOD.Cg-Tg (TcrαBDC2.5, TcrβBDC2.5)1Doi/DoiJ (BDC2.5; JAX 4460) TCR transgenic mice aged 5-8 weeks old or NOD.Cg-Tg(TcrαTcrβNY8.3)1Pesa/DvsJ (NY8.3; JAX 5868) TCR transgenic mice aged 5-12 weeks old. diabetic female13-week old NOD.shiltJ mice (NOD; JAX1976). For spontaneous diabetes development, female NOD mice were purchased at 3-4 weeks of age and monitored weekly for blood glucose by lateral vein bleeds using a glucometer test strip (Bayer Contour Next, and Bayer Contour Next EZ Glucometer). Mice with two consecutive readings at or above 250mg/dL were considered diabetic. 6–12-week-old NOD.Cg-Prkdcscid/J (NOD.*scid*; JAX 1303) females were the recipient mice for all T1D adoptive transfer studies.

### Cell Squeeze and Characterization of TACs

RBCs were isolated from whole blood of either C57BL/6 or NOD mice by Ficoll (GE 17-1440-03) gradient and were squeezed at 1x10^9^ RBCs/milliliters (mL) with various concentrations of antigens using a custom-made microfluidic squeeze device (Silex HT-10-022-70) at 60 PSI to generate TACs. TACs were washed with PBS to remove non-encapsulated antigens prior to injection or *in vitro* characterization.

For *in vitro* characterization of TACs, RBCs squeezed with 100µM OVA-647 (Invitrogen O34784) were stained with CD47 antibody or Isotype control antibody in FACS buffer (PBS + 2% FBS + 1mM EDTA). Cells were washed with Annexin V staining buffer and then stained with Annexin V (Biolegend 640945) according to the manufacturer’s protocol and assayed by flow cytometry.

### Assessment of TAC Clearance and Biodistribution

RBCs were isolated from C57BL/6 J mice and stained with 2µM PKH-26 dye (Sigma-Aldrich PKH26GL-1KT) according to the manufacturer’s instructions. Labeled RBCs were either left unprocessed or squeezed with 200μg/mL OVA Endofit™ (*In vivo*gen, vac-pova-100) to generate TAC-OVA. To assess TAC clearance, C57BL/6J mice were dosed with 1x10^9^ PKH26-labeled RBCs or PKH26-labeled TAC-OVA *via retroorbital* injection. Blood was collected at specific time points post injection by tail nick bleeds into tubes containing CPDA-1 (Sigma-Aldrich C4431). Immediately following collection, whole blood was assayed by flow cytometry to analyze PKH26^+^ RBCs.

For biodistribution studies, C57BL/6J mice were dosed with 1x10^9^ PKH-26-labeled TAC-OVA or PBS and liver, lung, bone marrow, lymph nodes and spleen were harvested 1hour post-dosing. Liver was perfused *via* the hepatic portal vein with 5mL of PBS followed by 3mL of 0.38% (w/v) collagenase XI (Sigma C7657-1G) in Hank’s Balanced Salt Solution (HBSS). Lung was perfused with PBS. The lung, spleen and lymph nodes (inguinal, cervical, and axillary) were digested for 15 minutes (min) at 37°C with 0.38% (w/v) collagenase XI in HBSS. Single cell suspensions were prepared, and cells from the spleen and lung were lysed in ammonium-chloride-potassium (ACK) lysis buffer (Gibco). The liver was digested for 30 min at 37°C with 0.38% (w/v) collagenase XI in HBSS and processed *via* manual dissociation. Hepatocytes were pelleted at 30 relative centrifugal force (rcf) for 4min and discarded, and RBC were lysed by treatment with ACK lysis buffer. Cells were washed, resuspended at 10x10^6^ live cells/mL, and mixed 1:1 with 33% percoll (GE 17-0891-01). Non-parenchymal cells were pelleted by centrifugation at 800 rcf for 30min. Single cell suspension from bone marrow were generated by flushing femurs and tibias with RPMI + 10% FBS + 1% Pen/Strep and RBCs were depleted by ACK lysis. PKH26 positive cells in various organs were analyzed by flow cytometry.

### OVA Immunization Model

For generating an immune response to OVA, C57BL/6J mice were dosed with 100μg Ovalbumin Endofit™ (*In vivo*gen) emulsified in a 1:1 mixture of Complete Freund’s Adjuvant (CFA; Sigma F5881) and Incomplete Freund’s Adjuvant (IFA; Sigma F5506) *via* subcutaneous route in the rear flank.

### Type 1 Diabetes Adoptive Transfer Models

Single cell suspensions from spleen and lymph nodes (pancreatic, inguinal, axillary, brachial, mesenteric, cervical) of either BDC2.5 or NY8.3 transgenic mice were resuspended in complete RPMI media (RPMI, 10% FBS, 1% Pen/Strep, 10mM HEPES, 55 μM beta-mercaptoethanol (βME), 2mM L-Glutamine) and plated at a concentration of 1x10^6^ live cells/ml in a 96-well U bottom plate. Cells were stimulated with respective cognate mimetope peptides, p31 (YVRPLWVRME for the BDC2.5 cells; Anaspec or NRPA7 (KYNKANAFL for the NY8.3 cells; Genscript) at a final concentration of 0.5μM for 4 days at 37°C and 5% CO2. After 4 days, cells were harvested from respective cultures and activation status and purity analyzed by flow cytometry.

NOD.*scid* mice were dosed by intravenous tail vein injection with either 5x10^6^ live BDC2.5 cells, 7x10^6^ live NY8.3 cells, or a co-transfer of either 2x10^6^ or 5x10^6^ each live BDC2.5 and live NY8.3 cells. For NY8.3 transfer, dead cells were removed by Ficoll gradient prior to transfer. Within two hours of adoptive transfer, animals were retro-orbitally injected with 1x10^9^ TACs either squeezed with PBS (empty TAC), 200μM HEL_11-25_, 200μM p31, 200μM NRPA7, or co-squeezed with 200μM each of p31 and NRPA7. Additional treatments with various TACs at a dose of 1x10^9^, were administered on specific days by retro-orbital injection.

For challenge with polyclonal diabetogenic cells, 10x10^6^ live cells isolated from spleen and lymph nodes (pancreatic, inguinal, axillary, brachial, mesenteric, cervical) of diabetic 13 week old NOD mice were adoptively transferred into NOD.*scid* recipients by intravenous tail vein injection.

Blood glucose levels in NOD.*scid* recipients were monitored daily for the first three weeks and thereafter twice weekly for the duration of the study. Blood glucose levels were measured from lateral vein bleeds as described for NOD mice. Mice with two consecutive readings at or above 250mg/dL over two consecutive days were considered to be diabetic.

### Preparation of Single-Cell Suspensions

Lymphocytes were isolated from spleen and lymph nodes by mechanical dissociation of tissue, followed by ACK lysis of splenocytes to remove RBCs. Lymphocytes were isolated from pancreata by initial incubation with 2mg/ml Collagenase Type IV (Worthington LS004189), 10μg/ml DNase I (Roche) in RPMI + 2% FBS + 1X Pen/Strep for 30 min at 37°C followed by ACK lysis.

### Flow Cytometry

Single cell suspensions were washed with PBS and stained with Live/Dead Fixable dye to assess viability (Thermo-Fisher L34957, L34955). For surface staining, cells were washed with FACs buffer, incubated for 5 min with Fc block (Miltenyi, 130-092-575) and stained with relevant antibodies diluted in FACs buffer for 20 min at room temperature. For tetramer staining, cells were pre-incubated for 30min at 4°C with relevant tetramers prior to the surface staining.

Intracellular staining of cytokines and transcription factors was performed with the eBioscience Foxp3/Transcription Factor Staining Buffer Set (00–5523–00) according to the manufacturer’s protocol. For intracellular IFNγ and T-bet staining, 8x10^6^ splenocytes were cultured for 5 hours in complete RPMI (10% FBS, 1% Pen/Strep) with p31 peptide at 2μg/ml. Golgi-plug and Golgi-stop (BD biosciences) was added for the last 4 hours of the culture.

For assessment of apoptosis, cells were first stained for activated caspase-3 with 500X dilution of FITC-VAD-FMK (Promega G7462) in RPMI + 10% FBS + 1% Pen/Strep at 37°C for 20 min.

All antibodies used for flow cytometry are listed in **Table S3**. All samples were acquired on Attune NxT Flow Cytometer (Thermo-Fisher). All flow cytometry data was analyzed using Flowjo v10.8.0 (BD).

### Pancreas Histology

Pancreata were collected and fixed in 4% PFA (Boston Bioproducts) for 24 hrs. Samples were then washed with PBS and shipped in 70% EtOH to Charles River Laboratories (CRL) for sectioning, staining and imaging. For histological analysis, multiple 5μm sections were stained with hematoxylin and eosin, imaged at 20X magnification and scored blindly for insulitis by a pathologist at CRL (Dr. Schantel A. Bouknight, DVM, PhD, DACVP). Degree of insulitis was scored on a grading scale based on the severity of mononuclear infiltration affecting the Islets of Langerhans. Grade 1: Infiltration of up to 25% of islet mass, ≤ 50% Islets of Langerhans affected. Grade 2: Infiltration of up to 25E% of islet mass, ≥ 50% Islets of Langerhans affected. Grade 3: Infiltration of up to 50% of islet mass, ≥ 50% Islets of Langerhans affected. Grade 4: Infiltration of up to 75% of islet mass, ≥ 50% Islets of Langerhans affected.

### IL-10 Depletion

5x10^6^ live BDC2.5 were adoptively transferred into NOD.*scid* mice. Mice were treated with TAC-p31 or TAC-HEL within two hours and on day two post adoptive transfer. Mice were dosed intraperitoneally with anti-IL-10 (clone JES5-2A5; Bio X Cell BE0049) or isotype control antibody (Bio X Cell BE0088) starting on day 7, 500μg of antibody was dosed on days 7, 9, and 11 post-transfer and then 2 times weekly through week 5.

### ELISPOT

3x10^5^ lymphocytes from spleen or dLN were restimulated with relevant antigens (2.5μM OVA protein, 1μM SIINFEKL, or 2mg/mL NRPA7) in defined serum-free media (Cellular Technology Limited CTLT-005) overnight at 37°C in a humidified chamber containing 5% CO2. Interleukin (IL) -2 (Cellular Technology Limited mIL2p-1M/10), Interferon gamma (IFNγ) (Cellular Technology Limited mIFNgp-2M/10), or Granzyme B (R&D Systems EL1865) ELISPOT were performed according to the manufacturers’ protocols. Spot forming units were enumerated by imaging and analysis with Immunospot Analyzer (Cellular Technology Limited).

### ELISA and Cytokine Bead Array

Lymphocytes from spleen or pancreas were cultured in complete DMEM media (DMEM + 10% fetal bovine serum, 1% Pen/Strep, 1% non-essential amino acids, 2mM L-Glutamine, 55μM βME) and restimulated with relevant antigens (2.5μM OVA protein, 2μg/mL p31, 2μg/mL NRPA7). IL-2, IFNγ, TNFα, IL-17A, and IL-22 production was assayed by multi-analyte cytokine bead array (CBA) after 24h (Biolegend 740818, 741043). IL-10 levels in supernatant were assayed after 72h of stimulation by IL-10 ELISA (Biolegend 431414). For CBA assays from T1D studies, 5x10^5^ cells per well resuspended in 100μL of relevant stimuli was used. For CBA assays in OVA study, the cells were plated at 4x10^6^ cells/well in 200μL stimulation media.

### Statistical Analysis

Statistical analysis was calculated by two-tailed Mann-Whitney U test between control group and the treatment group when only a single pair of conditions was analyzed. For statistical analysis of more than two groups, ordinary one-way ANOVA was utilized, and multiple comparisons were analyzed by Tukey’s multiple comparisons test. For disease incidence, Log-rank (Mantel-Cox) test was used to compare treatment and control groups. Statistics were calculated in Prism 9 (GraphPad). *P* values < 0.0001 are summarized as ***, otherwise exact *P* values are reported.

## Results

### TACs Have a Modified Membrane and Are Primed for Uptake by APCs in the Reticuloendothelial System (RES)

**Figure 1:**
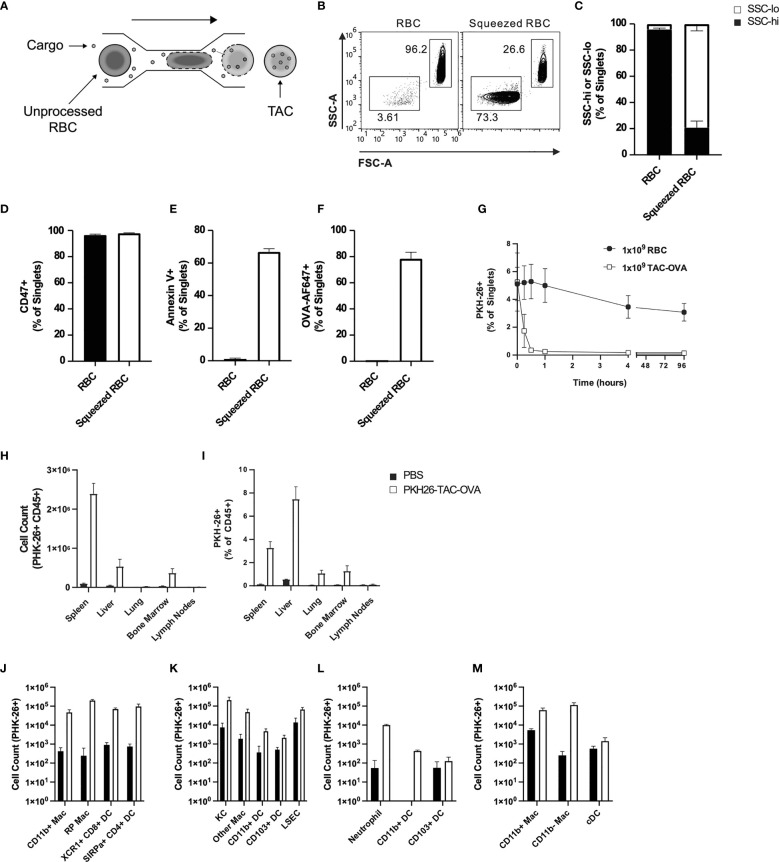
TACs are Annexin V positive and are primarily engulfed by macrophages in the spleen and liver. **(A)** Schematic of microfluidic platform for squeezing RBC with target antigen to generate TAC. **(B–F)** Flow cytometry characterization of untouched RBC or RBC squeezed with OVA-647. **(B)** Representative and **(C)** quantitative flow cytometry of light scatter by untouched or squeezed RBC. Quantification of **(D)** CD47 and **(E)** Annexin V staining of and **(F)** OVA-647 delivery to squeezed versus untouched RBC. **(B–F)** are gated on singlets. **(G–M)** RBC squeezed with OVA (TAC-OVA) labeled with PKH-26 or controls (PKH-26^+^ RBC or PBS) were injected into C57BL/6 mice. **(G)** Frequency of peripheral blood singlets PKH-26^+^ at indicated timepoints post-injection. **(H)** Total count of PKH-26^+^ CD45^+^ cells and **(I)** frequency of PKH-26^+^ among CD45^+^ leukocytes cells in the indicated organs one-hour post-injection. **(J-M)** Uptake of PKH26^+^ TACs by various APC subsets in the **(J)** spleen, **(K)** liver, **(L)** lungs and **(M)** bone marrow one-hour post-injection. OVA squeeze concentration was 100mM **(B–F)** and 5mM for **(G–M)**. n=3 and representative of at least two independent experiments. Mean +/- SD.

Previously, the microfluidic Cell Squeeze^®^ platform has been shown to efficiently deliver a broad array of materials into diverse cell types (27, 30). Here, we applied the Cell Squeeze technology to engineer RBCs with certain characteristics that resembled aged RBCs and to be efficiently loaded with cargo. To this end, we investigated several microfluidic chip designs and squeeze parameters utilizing mouse RBCs and fluorescently labeled Ovalbumin (OVA-647) (**Figure 1A**). We identified a proprietary microfluidic chip design and squeeze conditions that generated a cell population with low Forward Scatter (FSC) and Side Scatter (SSC) profile (FSC^lo/^SSC^lo^) reminiscent of aged RBCs that undergo cell shrinkage during eryptosis (**Figures 1B, C**). Unprocessed RBCs were dominated by FSC^hi^/SSC^hi^ cell population (**Figures 1B, C**). Eryptotic or senescent RBCs are characterized by exposure of the classical phagocytic signal, phosphatidyl serine (PS), on the cell surface (31). Additionally, the expression of CD47, an anti-phagocytic marker, is known to be reduced on eryptotic RBCs (12, 13, 32). Flow cytometry revealed higher exposure of PS in squeezed RBCs as measured by Annexin V staining but no alteration in CD47 levels compared to control RBCs (**Figures 1D, E**). Furthermore, the squeezed RBCs were efficiently loaded with OVA (78 ± 5% in squeezed RBCs vs 0.2 ± 0.1% in un-squeezed RBCs) (**Figure 1F** and **Figure S1A-C**). RBCs engineered using the Cell Squeeze platform that are FSC^lo^/SSC^lo^, have externalized PS and encapsulated cargo are hereafter referred to as TACs.

RBCs in mice have a lifespan of 45 days (33). To determine if the squeezed RBCs would be cleared faster from circulation due to higher PS levels, we intravenously administered unprocessed RBCs or RBCs squeezed with OVA (TAC-OVA) that were labeled with fluorescent dye PKH26 into C57BL/6 mice. Flow cytometry analysis showed that there was no significant change in the percentage of PKH26- labeled RBCs throughout time course of the study (**Figure 1G**). In contrast, labeled TACs were rapidly cleared from the circulation with a half-life of 8 min (**Figure 1G**).

Aged RBCs are cleared from circulation by phagocytes in the RES (34–36). Due to the similarity between eryptotic RBCs and TACs, we asked whether TACs were cleared in a similar fashion in the RES. To test this, PKH26-labeled TAC-OVA was administered in mice and various organs in the RES harvested 1 hour (hr) after dosing. Mice injected with PBS served as negative control. Flow cytometry analysis revealed uptake of PKH26-labeled TAC-OVA in CD45^+^ cells in the spleen, liver, lung and bone marrow but not in the lymph nodes (**Figures 1H, I**). The majority of PKH26-labeled cells were seen in spleen and liver with the spleen displaying the highest absolute counts of PKH26-labeled cells (**Figure 1H**). We next determined which cell populations in the various organs were primarily responsible for engulfing TACs. In the spleen, predominant uptake of PKH26-labeled TAC-OVA was by red pulp macrophages (RPMs) and to a lesser extent by XCR1^+^ cross-presenting DCs, SIRPα^+^ DCs and CD11b^+^ macrophages (**Figure 1J**). Analysis of the liver subsets revealed the highest number of PKH26-labeled cells in Kupffer cells (KCs) followed by liver sinusoidal endothelial cells (LSECs) and other macrophages (**Figure 1K**). In the lung, uptake was seen in neutrophils and Cd11b+ DCs (**Figure 1L**) and bone marrow uptake was carried out by macrophages (**Figure 1M**). Taken together, these results demonstrate that TACs are cleared by the same cell populations and organs involved in the removal of eryptotic RBCs.

### TACs Loaded With Ovalbumin Suppress Antigen-Specific Endogenous T Cell Responses *via* a Spleen Independent Manner

**Figure 2:**
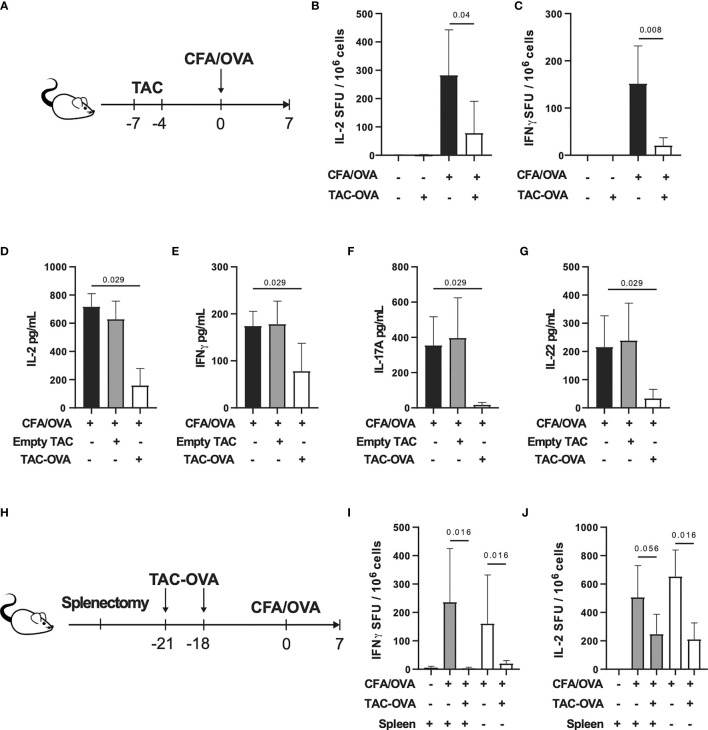
TAC-OVA suppresses endogenous immune responses to OVA challenge irrespective of the spleen. **(A-G)** C57BL/6 mice were untreated or treated with TAC squeezed with PBS (Empty TAC) or OVA (TAC-OVA) on days -7 and -4. Mice were immunized on day 0 with CFA and OVA. 7 days later, immune responses were assessed in the draining LNs. **(B)** Number of IL-2 and **(C)** IFNγ producing cells were assayed by ELISpot after restimulation with **(B)** OVA protein or **(C)** SIINFEKL peptide. **B-C** are n = 3 for naïve and n=5 for other groups. Secretion of **(D, E)** Th1 and **(F, G)** Th17 associated cytokines after OVA protein restimulation. **(D–G)** show n=4 pools of 2 mice each per group. **(H-J)** C57BL/6 mice underwent sham surgery or splenectomy. Mice were either untreated or treated with TAC-OVA and challenged with OVA in CFA. OVA-specific immune responses were assessed in the draining LNs after 7 days by ELISpot. **(I)** IFNγ and **(J)** IL-2 producing cells after restimulation with **(I)** SIINFEKL peptide or **(J)** OVA protein. **(I-J)** n = 4-5 per group. **(A-J)** TAC dose was 1x10^9^ and OVA squeeze concentration was 100μM. Mean +/- SD. Mann-Whitney U Test. Representative of at least 2 independent experiments.

To determine the ability of TACs to tolerize against endogenous antigen-specific T cell responses, we treated mice with two doses of TACs encapsulating OVA (TAC-OVA), 7 and 4 days prior to challenge with OVA/CFA as shown in **Figure 2A**. 7 days post-immunization, draining lymph nodes (dLNs) were analyzed for antigen-specific T cell responses after *ex vivo* stimulation with OVA protein or SIINFEKL peptide. ELISpot analysis revealed a 3.6- and 7.4-fold reduction in Interleukin (IL)-2 and Interferon-gamma (IFNγ) producing OVA-specific T cells, respectively, in TAC-OVA treated animals compared to controls (**Figures 2B, C**). The suppression of cytokine responses to SINFEKL peptide stimulation correlated with decreased frequency of OVA-specific CD8 T cells by Class I MHC tetramer staining (**Figure S2**). Reduction in OVA-specific T cell responses were also seen in spleen (**Figures S3A, B**).

Repeated administration of soluble protein has been shown to induce tolerance (37, 38). To determine if repetitive administration of soluble protein can confer the same degree of tolerization as TACs, mice were treated with three doses of soluble OVA or TAC-OVA, 7, 4 and 1 day prior to immunization with OVA/CFA. The concentration of OVA administered as free protein or encapsulated in TACs were equivalent. On day 7 after immunization, antigen-specific T cell responses were analyzed in dLNs after *ex vivo* stimulation with either OVA protein or SINFEKL peptide. ELISpot analysis revealed that TAC-OVA treatment was more effective at suppressing both IFNγ and IL-2 secretion from OVA-specific T cells compared to soluble OVA (**Figure S4**).

Clearance of eryptotic RBCs and apoptotic cells by efferocytosis induces an anti-inflammatory response (39). Since TACs were engineered to resemble eryptotic RBCs that are cleared in a tolerogenic manner, we wanted to rule out that efferocytosis alone is not sufficient for inducing antigen-specific tolerance. To test this, we administered empty TAC squeezed without antigen or TAC-OVA and thereafter challenged mice with OVA/CFA as shown in **Figure 2A**. On day 7, after antigenic challenge, lymphocytes from dLNs were analyzed for T helper (Th) 1 and Th17 cytokine responses after stimulation with OVA *ex vivo*. TAC-OVA led to 4.5-, 2.2-, 21.6- and 6.5-fold reduction in IL-2, IFNγ, IL-17 and IL-22, respectively, compared to empty TACs (**Figures 2D–G**). Levels of Th1 and Th17 cytokines in empty TAC treated mice were similar to immunized mice that were untreated. These data demonstrate that presentation of antigen from TACs in a non-inflammatory context is critical for antigen-specific tolerance induction.

Since TACs are cleared by phagocytes in the spleen and liver, we next sought to determine the relative contributions of these organs in TAC-mediated T cell tolerance. Mice that underwent either sham surgery (sham) or splenectomy (splenectomized) were dosed with TAC-OVA and challenged with OVA/CFA as shown in **Figure 2H**. Antigen-specific immune responses were evaluated from dLN following *ex vivo* restimulation with OVA. ELISpot analysis showed repression of OVA-specific IL-2 and IFNγ from splenectomized mice at comparable levels to sham control mice (**Figures 2I, J**). These results indicate that the spleen is dispensable for TAC-induced T cell tolerance.

### Treatment With TACs Loaded With p31 Delays BDC2.5 T Cell Induced Diabetes by Reducing T Cell Infiltration and Proinflammatory Cytokine in Pancreas

**Figure 3:**
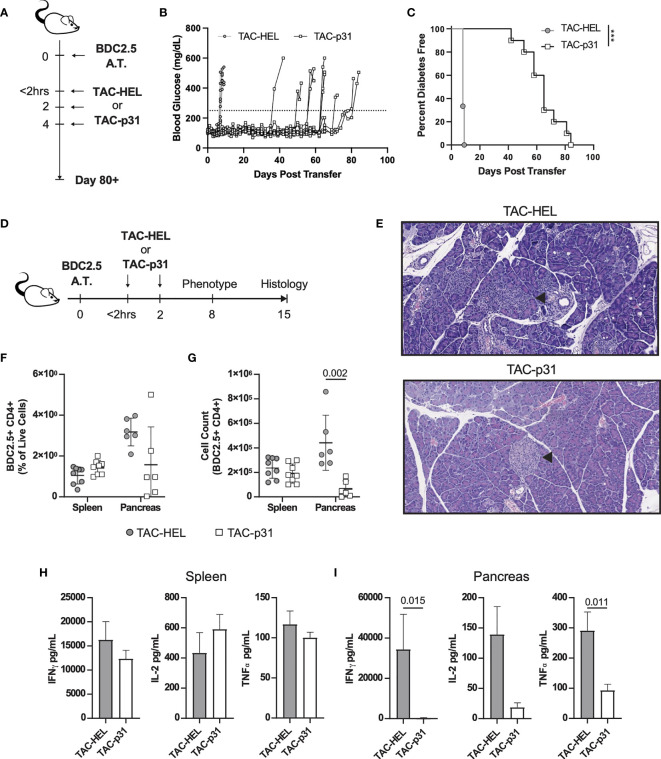
TAC-p31 impairs pancreatic islet infiltration and BDC 2.5 effector function. **(A-C)** 5x10^5^ Activated BDC2.5 cells were transferred into NOD.*scid* mice on day 0. Mice were treated with TAC-HEL (control) or TAC-p31 within 2 hours and on days 2 and 4 post-transfer and monitored for 80+ days. **(B)** Blood glucose and **(C)** diabetes disease incidence of mice treated with TAC-HEL or TAC-p31. **(B, C)** Disease was defined as two consecutive days ≥250mg/dL (dotted line). TAC-HEL n = 9, TAC-p31 n = 10. Log-rank (Mantel-Cox) test. **(D-I)** Activated BDC2.5 cells were transferred into mice on day 0. Mice were treated with TACs within 2 hours and on day 2. **(E)** Representative H&E staining of pancreata on day 15 post transfer. Arrowhead indicates Islet of Langerhans. n = 2 per group. **(F)** Frequency and **(G)** number of BDC2.5^+^ Cells (CD90.2^+^ CD4^+^ BDC2.5 Tetramer^+^) in spleens and pancreata of mice on day 8. **(H-I)** Th1 Cytokine secretion by p31 stimulated cells from the **(H)** spleen and **(I)** pancreas on day 8. **(F–I)** show samples of cells pooled from 2-5 animals. Spleen n = 10, pancreas n=6 pools per group. Mean +/- SD. Mann-Whitney U Test. Data from 2 independent experiments. **(A-I)** TAC dose was 1x10^9^ and peptide squeeze concentration was 200μM. ***P < 0.0001.

To evaluate whether TACs could induce antigen-specific tolerance in a pathologic setting, we utilized an aggressive adoptive transfer system in which diabetes was induced by transfer of activated BDC2.5 TCR transgenic (40, 41) T cells into NOD.*scid* recipients that are deficient in B and T cells. BDC2.5 transgenic CD4 T cells are reactive against epitopes from islet autoantigen chromogranin or the peptide mimetope p31 (42, 43). Prior to adoptive transfer into NOD.*scid* mice, BDC2.5 T cells were activated by stimulating with p31 mimetope *in vitro* and purity was analyzed by flow cytometry (**Figure S5**). Mice were treated with either TACs encapsulating an irrelevant peptide HEL_11-25_ (TAC-HEL) or TACs loaded with p31 within 2 hrs and on days 2 and 4 post-adoptive transfer as shown in **Figure 3A**. Treatment with TAC-p31 delayed onset and incidence of hyperglycemia by a median of 65 days with complete prevention of diabetes up to 43 days post adoptive transfer (**Figures 3B, C**). In contrast, all TAC-HEL treated mice developed hyperglycemia within 7 days (**Figures 3B, C**).

We next performed histology on pancreata from control or TAC-p31 treated mice on day 15 post-transfer as depicted in **Figure 3D**. Pancreata from TAC-HEL treated mice had high severity of insulitis characterized by marked numbers of infiltrating mononuclear cells and areas of islet cell degeneration (**Figure 3E** *top*, **Table S1**). In contrast, pancreata from TAC-p31 treated mice had normal islet architecture and minimal infiltration by mononuclear cells (**Figure 3E** *bottom*, **Table S1**).

To explore the mechanisms underlying reduced pancreatic infiltration and preservation of islets upon TAC-p31 treatment, we examined BDC2.5 T cells and their effector functions in spleen and pancreas on day 8 after transfer. The frequency of BDC2.5 CD4^+^ T cells were reduced 2-fold in the pancreas of TAC-p31 compared to TAC-HEL treatment (**Figure 3F**). The total numbers of BDC2.5 CD4^+^ T cells showed a marked reduction with approximately (~) 8-fold decrease in the pancreas after treatment with TAC-p31 compared to TAC-HEL (**Figure 3G**). Strikingly, the percentages and absolute numbers of BDC2.5 CD4^+^ T cells remained elevated in the spleen and were comparable between TAC-HEL and TAC-p31 treatments (**Figures 3F, G**). These results suggest that TAC-p31 treatment impairs trafficking of BDC2.5 T cells to the islets leading to their retention in the spleen. Correspondingly, Th1 cytokine secretion by *ex vivo* p31 peptide stimulated lymphocytes from TAC-p31 treated animals showed 126-, 11.4- and 7.4-fold reduction in IFNγ, IL-2 and Tumor Necrosis Factor (TNF) -α, respectively, in the pancreas compared to TAC-HEL treated animals, which displayed increased levels of these cytokines. (**Figure 3H**). In contrast, Th1 cytokines in spleen remained elevated in TAC-p31 treated animals with levels comparable to mice that received TAC-HEL treatment (**Figure 3I**).

### TAC-p31 Treatment Increases Antigen-Specific Tregs and Augments IL-10 Production

**Figure 4:**
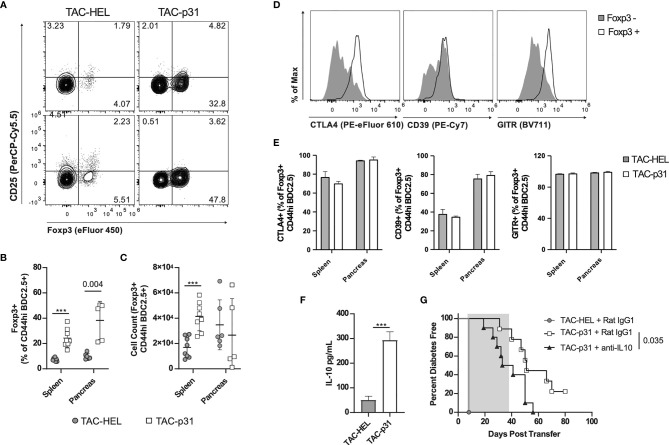
p31 loaded TACs increase BDC2.5 Tregs and IL-10 production. **(A-G)** NOD.*scid* mice were dosed as described in **Figure 3D** and **(A-F)** phenotype was assayed on day 8. **(A)** Representative and **(B, C)** quantitative flow cytometry of Foxp3^+^ Tregs among CD44^hi^ BDC2.5 cells (CD90.2^+^ CD4^+^ BDC2.5 Tetramer^+^ CD44^hi^) in the spleen **(A **top**)** and pancreas **(A **bottom**)**. Cells were pooled from 2-5 animals. Spleen n=8, pancreas n=5-6 pools per group. Data from 2 independent experiments. **(D)** Representative flow cytometry histograms of CTLA-4, CD39 and GITR among Foxp3^+/-^ CD44^hi^ BDC2.5 cells in the pancreas of a TAC-p31 treated animal. **(E)** Frequency of CTLA-4, CD39 and GITR among Foxp3^+^ CD44^hi^ BDC2.5 cells in the indicated organs. n=3 pools of cells from 3-4 animals. Representative data from two independent experiments. **(F)** Production of IL-10 by splenocytes after 72-hour p31 stimulation. Cells were pooled from 2 animals, 10 pools per group. Data is combined from two independent experiments. **(G)** Diabetes incidence among mice treated with anti-IL-10 or isotype control (Rat IgG1) antibody starting on day 8 post-transfer for 4 weeks (shaded region). n=10 mice per group. Log-rank (Mantel-Cox) test. **(A-F)** Show mean +/- SD. Mann-Whitney U Test. **(A-G)** TAC dose was 1x10^9^ and peptide squeeze concentration was 200μM. ***P < 0.0001.

Regulatory T cells play a critical role in controlling autoreactive T cells *via* various suppressive mechanisms (44, 45). To investigate whether the above reduction in BDC2.5 effector responses were due to an increase in Tregs, we performed flow cytometry to evaluate Tregs in spleen and pancreas. In the BDC2.5 adoptive transfer model, only half the Tregs express the bona fide Treg makers CD25 and the transcription factor Foxp3, whereas the other half is characterized by expression of Foxp3 alone (46). In the current transfer model, all BDC2.5 Tregs expressed Foxp3 but had downregulated CD25 irrespective of TAC treatment (**Figure 4A**). The percentage of BDC2.5 Foxp3^+^ Tregs was increased 3.3- and 4.4-fold in spleen and pancreas, respectively, in the TAC-p31 treated animals compared to TAC-HEL (**Figure 4B**). However, the increase in absolute numbers of BDC2.5 Tregs was only observed in the spleen but not in the pancreas of TAC-p31 treated animals (**Figure 4C**), which is due to the marked reduction in the numbers of BDC2.5 T cells in the pancreas. Examination of Treg functional markers revealed elevated expression of CTLA4, CD39 and GITR on Foxp3^+^ BDC2.5 Tregs but there was no difference in the expression levels of these markers between TAC-HEL and TAC-p31 treatments (**Figures 4D, E**).

Splenocytes stimulated *ex vivo* with p31 peptide produced 5.8-fold higher levels of IL-10 in the TAC-p31 treatment compared to controls (**Figure 4F**). To determine if IL-10 plays a role in TAC-p31 induced tolerance, activated BDC2.5 T cells were adoptively transferred into NOD.*scid* recipients and were first treated with two doses of either TAC-HEL or TAC-p31 as described above. Subsequently, recipient mice were administered either IL-10 blocking or an isotype control (rat IgG) antibody on days 7, 9, 11 and then twice a week up to 5 weeks post-transfer. The incidence of diabetes development was accelerated by blockade of IL-10 compared to isotype control treatment (**Figure 4G**). Altogether, these results suggest that TAC-p31 increases antigen-specific Tregs and suppressor cytokine production that modulates the pathogenicity of BDC2.5 T cells.

### TAC-p31 Increases Distinct Antigen-Specific Treg Subsets

**Figure 5:**
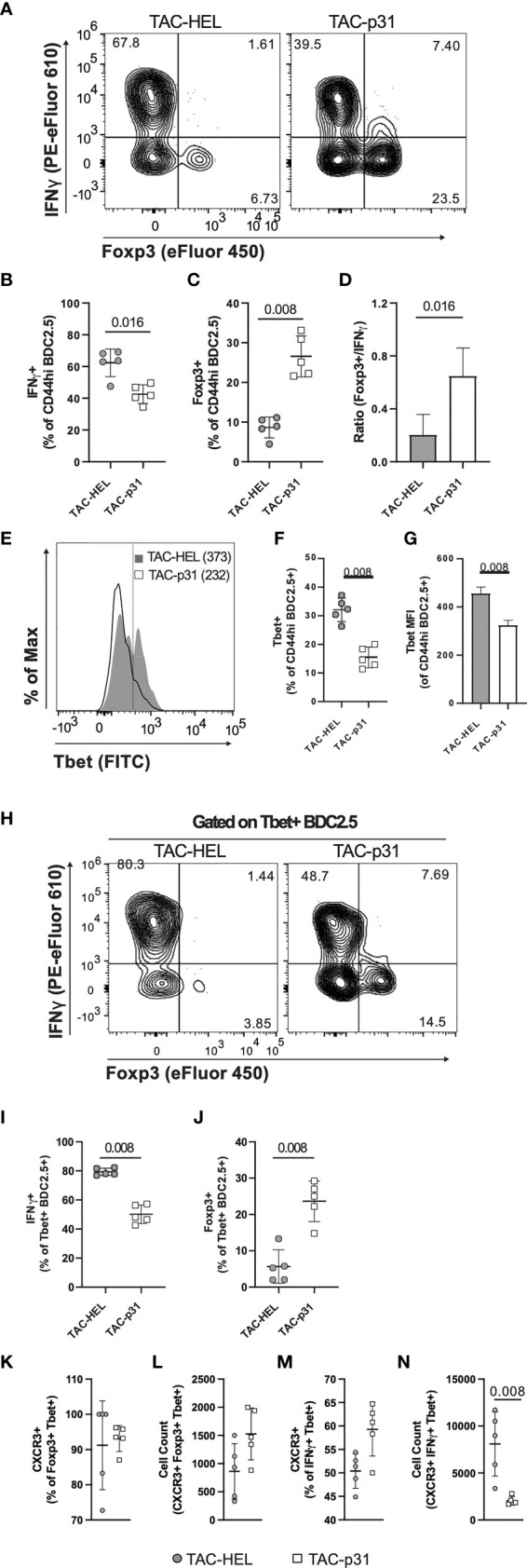
TAC- p31 alters the ratio of BDC2.5 Tregs to Teffs and reduces CXCR3^+^ Teffs. **(A-N)** Mice were dosed as described in **Figure 3D** and phenotype of splenocytes stimulated with p31 was assayed 8 days post-transfer. **(A)** Representative and quantitative flow cytometry of frequency **(B)** IFNγ^+^ and **(C)** Foxp3^+^ among CD44^hi^ BDC2.5 cells (CD90.2^+^ CD4^+^ BDC2.5 Tetramer^+^ CD44^hi^). **(D)** Ratio of number of Foxp3^+^/number of IFNγ^+^ CD44^hi^ BDC2.5 cells. **(E)** Representative and quantitative flow cytometry of Tbet **(F)** frequency and **(G)** expression (median fluorescence intensity, MFI) among CD44^hi^ BDC2.5 cells. **(H)** Representative and quantitative flow cytometry of **(I)** IFNγ^+^ and **(J)** Foxp3^+^ frequency among Tbet^+^ CD44^hi^ BDC2.5 cells. **(K)** Frequency and **(L)** absolute counts of CXCR3 expressing Foxp3^+^ Tbet^+^ CD44^hi^ BDC2.5 Treg. **(M)** Frequency and **(N)** absolute counts of CXCR3 expressing IFNγ^+^ Tbet^+^ CD44^hi^ BDC2.5 Teff. **(A-N)** TAC dose was 1x10^9^ and peptide squeeze concentration was 200μM. Each group is five pools of two animal each. Mean +/- SD. Mann-Whitney U Test. Representative of two independent experiments.

Increased numbers of BDC2.5 Tregs despite elevated numbers of BDC2.5 effectors in the spleen suggested that TAC-p31 might alter the balance between Tregs and effector T cells (Teffs). Intracellular cytokine staining on day 8 post adoptive transfer revealed a modest 1.5-fold decrease in IFNγ producing Teffs and 3.2-fold increase in Foxp3^+^ Tregs in the splenic BDC2.5 T population after TAC-p31 treatment (**Figures 5A–C**). Overall, there was a 3-fold increase in the ratio of Foxp3^+^ Tregs to IFNγ^+^ Teffs in the TAC-p31 treated mice (**Figure 5D**).

We next evaluated T-bet expression as it controls expression of the hallmark Th1 cytokine IFNγ. There was only a modest downregulation in T-bet expression but the frequency of T-bet positive BDC2.5 effectors was decreased by 2.2-fold after TAC-p31 treatment compared to controls (**Figures 5E–G**). In addition to Th1 cells, a subset of Tregs co-express Foxp3 and T-bet that have unique migratory and suppressive functions during type-1 inflammation (47). TAC-p31 treatment led to only a nominal decrease in the frequency of T-bet^+^ IFNγ^+^ cells whereas the frequency of T-bet^+^ Foxp3^+^ T cells was increased 6.2-fold compared to TAC-HEL treatment indicating a shift in the balance of these specialized suppressor T cells (**Figures 5 H–J**).

T-bet directly induces the expression of CXC chemokine receptor 3 (CXCR3) in Th1 cells as well as a subset of Tregs that co-express T-bet and Foxp3 and controls their migration (47, 48). The accumulation of BDC2.5 effectors in the spleen led us to hypothesize that reduced T-bet expression after TAC-p31 treatment would alter CXCR3 expression. To test this hypothesis, we performed flow cytometry to evaluate expression of CXCR3 in BDC2.5 effectors and Tregs. There was no significant difference in the percentage of CXCR3 expressing effectors and Tregs between the two treatment groups (**Figures 5K, M**). However, there was a 4.5-fold reduction in the absolute numbers of CXCR3^+^ BDC2.5 effectors and a modest 1.8-fold increase in CXCR3+ Tregs in TAC-p31 compared to TAC-HEL treatment (**Figures 5L, N**). Taken together, these results suggest that alterations in Treg subsets with distinct functions maybe a key driver for antigen-specific tolerance induced by TAC-p31.

### TACs Loaded With NRPA7 Induce Potent Deletion of NY8.3 T Cells and Prevents Transfer of Diabetes

**Figure 6:**
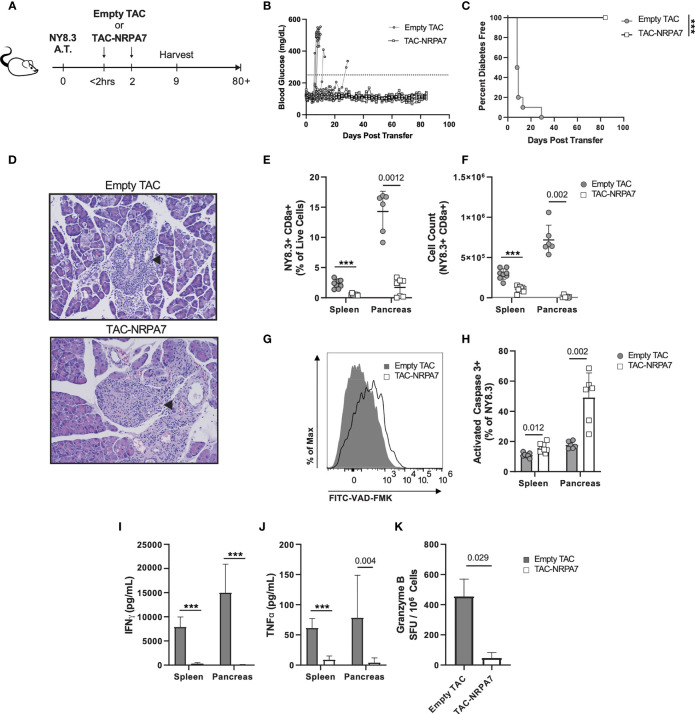
TAC-NRPA7 prevents diabetes induced by NY8.3 T cells by deleting antigen-specific T cells. **(A-K)** 7x10^6^ activated NY8.3 cells were transferred into NOD.*scid* mice on day 0. 1x10^9^ empty TAC or TAC-NRPA7 (200μM squeeze) were dosed within 2 hours and on day 2, and **(E-K)** phenotype was assessed on day 9. **(B)** Blood glucose and **(C)** diabetes disease incidence of mice treated with empty of NRPA7 loaded TACs. **(B, C)** Disease was defined as two consecutive days ≥250mg/dL (dotted line). n = 10 per group. Log-rank (Mantel-Cox) test. **(D)** Representative H&E staining of pancreata. Arrowhead indicates islet of Langerhans. n = 2 per group. **(E)** Frequency and **(F)** number of NY8.3 cells (CD90.2^+^ CD8α^+^ NRPA7 tetramer^+^) in the indicated organs. **(G)** Representative activated caspase 3 (FITC-VAD-FMK) staining of splenocytes and **(H)** quantification in the indicated organs. Gated on NY8.3 cells irrespective of fixable viability stain. **(I)** IFNγ and **(J)** TNFα secretion by NRPA7 stimulated cells from indicated organs. **(E-J)** Spleen n = 7-9, pancreas n = 6 pools per group. Data from 2 independent experiments.**(K)** ELISpot of Granzyme B producing splenocytes after stimulation with NRPA7 peptide. n = 4 pools per group. **(E-K)** Each symbol is a pool of 2-4 animals. Mean +/- SD. Mann-Whitney U Test. **(A-K)** ***P < 0.0001.

We next sought to determine whether TACs can prevent T1D induced by transfer of diabetogenic CD8 T cells given the importance of these cells in destruction of beta cells. To this end, NY8.3 CD8 transgenic T cells specific for IGRP were stimulated *in vitro* with mimetope peptide NRPA7 (49) and assessed for purity by flow cytometry (**Figure S6**). Thereafter activated NY8.3 CD8 T cells were adoptively transferred into NOD.*scid* recipients and either treated with empty TACs or TACs encapsulating NRPA7 as shown in **Figure 6A**. TAC-NRPA7 completely prevented hyperglycemia and T1D onset whereas treatment with control TACs offered no protection (**Figures 6B, C**). Histological analysis of pancreata were performed on day 9 post-adoptive transfer. In control treated animals, islets of Langerhans were completely obscured by mononuclear infiltrates and difficult to detect as they were markedly decreased in size (**Figure 6D** *top*, **Table S2**). In contrast, pancreas of TAC-NRPA7 treated animals had mild infiltration of mononuclear cells and overall lower severity of insulitis (**Figure 6D** *bottom*, **Table S2**).

To determine the mechanisms by which TAC-NRPA7 induces antigen-specific tolerance, flow cytometric analysis was performed on spleen and pancreata in both treatment groups on day 9 post-transfer. The percentages and absolute numbers of NY8.3 T cells were significantly reduced in both spleen and pancreas after TAC-NRPA7 treatment compared to empty TAC treatment (**Figures 6E, F**). Notably, the effect of TAC-NRAP7 was striking in the pancreas with 14-fold and ~54-fold reduction in percentage and absolute numbers, respectively, of NY8.3 T cells compared to controls. To investigate the mechanisms underlying the profound reduction in NY8.3 T cells in target organs, we asked whether TAC-NRAP7 led to deletion of pathogenic CD8 T cells. NY8.3 T cells undergoing apoptosis was assessed by staining for activated caspase 3. TAC-NRPA7 treatment led to increased apoptosis of NY8.3 T cells in both spleen and pancreas but the frequency of apoptotic NY8.3 T cells in pancreas was 3-fold higher than in the spleen (**Figures 6G, H**). Splenocytes and pancreatic lymphocytes restimulated with NRPA7 peptide *in vitro* showed significant impairment in proinflammatory cytokine secretion in TAC-NRPA7 treated animals consistent with their reduced NY8.3 T cell numbers (**Figures 6I, J**). In the spleen, TAC-NRPA7 treatment led to ~27- and 7-fold reduction in IFNγ and TNF-α, respectively compared to empty TAC-treatment. The effect of TAC-NRPA7 was profound in the pancreas with a 375- and 19.6-fold reduction in IFNγ and TNF-α, respectively, compared to control treatments. Furthermore, the number of granzyme B producing NY8.3 T cells as determined by ELISpot was reduced 7-fold in TAC-NRPA7 treated mice compared to controls (**Figure 6K**). Together, these data demonstrate that the major mechanism of antigen-specific tolerance induced by TAC-NRPA7 is deletion of autoreactive CD8 T cells leading to reduced pancreatic infiltrates, proinflammatory cytokines, and cytotoxic mediators.

### TAC-p31 Increases Antigen-Specific Tregs That Exert Bystander Suppression

**Figure 7:**
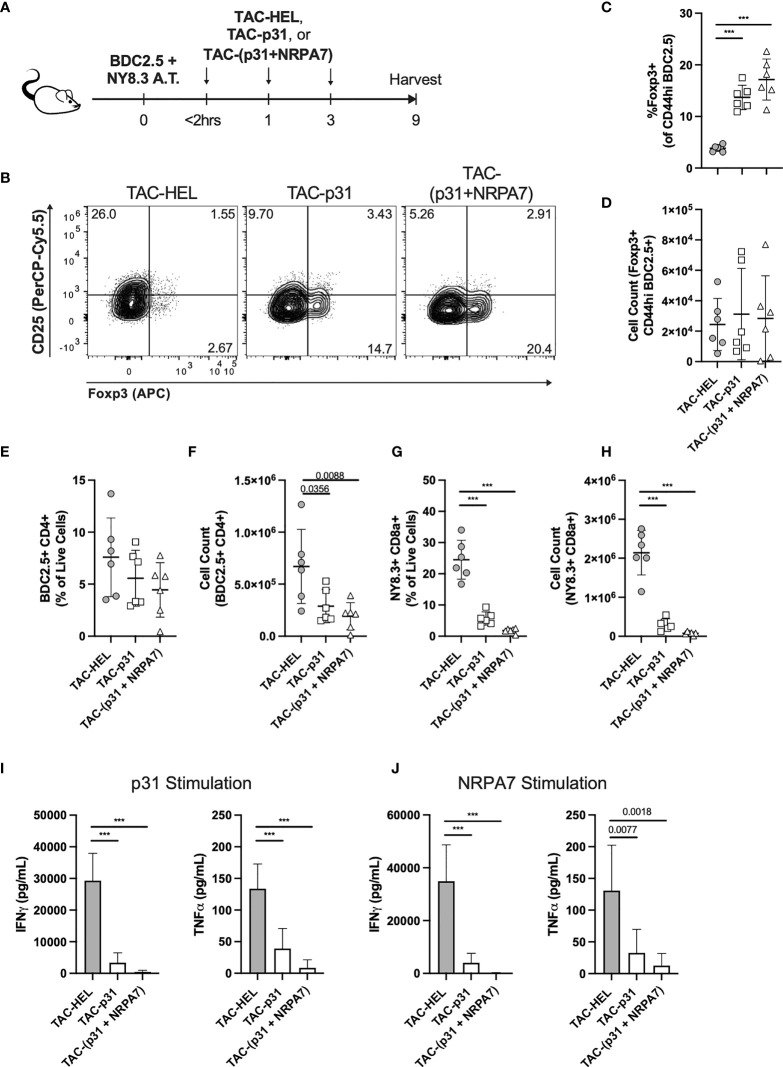
Treatment with TAC-p31 alone increases BDC2.5 Tregs that suppress NY8.3 CD8^+^ T cells *via* bystander suppression. **(A-J)** 2x10^6^ each activated BDC2.5 and NY8.3 T cells were co-transferred into NOD.*scid* mice and treated with TAC-HEL (control), TAC-p31 or TAC-(p31+NRPA7), and T cell phenotype was assessed in the pancreas on day 9 post-transfer. **(B)** Representative and **(C, D)** quantitative flow cytometry of Foxp3^+^ Tregs among CD44^hi^ BDC2.5 cells (CD90.2^+^ CD4^+^ BDC2.5 Tetramer^+^ CD44^hi^) among the indicated treatment groups. **(E)** Frequency and **(F)** total number of pancreatic infiltrating BDC2.5 CD4^+^ T cells (CD90.2^+^ CD4^+^ BDC2.5 Tetramer^+^) in the indicated TAC treatment groups. **(G)** Percentage and **(H)** total number of pancreatic infiltrating NY8.3 CD8^+^ T cells (CD90.2^+^ CD8a^+^ NY8.3 Tetramer^+^) in the indicated TAC treatment groups. Proinflammatory cytokine secretion by **(I)** BDC2.5 and **(J)** NY8.3 cells after stimulation with **(I)** p31 or **(J)** NRPA7 peptide by cells from the indicated treatment groups. **(B-J)** TAC dose was 1x10^9^ and squeeze concentration was 200μM each peptide. Each symbol is a pool of 3-5 animals. n=6 pools per group. Mean +/- SD. One-way ANOVA with Tukey’s multiple comparisons test. ***P < 0.0001.

A key challenge in autoimmune disease is the presence of polyclonal autoreactive T cells and epitope spreading. Treg mediated bystander suppression is key for controlling epitope spreading. To evaluate whether antigen-specific Tregs elicited upon TAC treatment could exert bystander tolerance, we co-transferred 2x10^6^ each activated p31-specific BDC2.5 CD4 and NRPA7-specific NY8.3 CD8 transgenic T cells into NOD.*scid* mice. Purity of transferred T cells were evaluated by flow cytometry (**Figure S7**). Recipients were thereafter treated with three doses of TACs encapsulating HEL or p31 or co-encapsulating p31 and NRPA7 as depicted in **Figure 7A**. On day 9 post-transfer, pancreatic lymphocytes were isolated for analysis by flow cytometry. The percentages of Foxp3^+^ BDC2.5 Tregs were significantly elevated with a 3.6- and 4.5-fold increase in TAC-p31 and TAC (p31+NRPA7) treatments, respectively, compared to controls but the absolute numbers were unaltered (**Figures 7B–D**). Treatment with TACs encoding autoantigens led to only a modest decrease in frequency of BDC2.5 T cells but a 2 to 4-fold reduction in their total numbers compared to control treatment (**Figures 7E, F**). We next evaluated whether BDC2.5 Tregs expanded after TAC-p31 treatment could suppress NY8.3 CD8 T cells in a bystander manner. Control treated mice displayed high frequency and numbers of NY8.3 T cells whereas treatment with TAC-p31 alone led to a 4.3- and 7-fold reduction in percentages and absolute numbers of NY8.3 CD8 T cells, respectively, indicating bystander suppression (**Figures 7G, H**). TAC (p31+NRPA7) treatment had a more pronounced effect with a 13.7- and 28.6-fold reduction in NY8.3 T cell frequency and total cell numbers, respectively, compared to controls (**Figures 7G, H**).

To further demonstrate bystander activity by BDC2.5 Tregs after TAC-p31 treatment, we evaluated effector responses of BDC2.5 and NY8.3 T cells after restimulation with p31 and NRPA7 peptide, respectively. Proinflammatory cytokine secretion by BDC2.5 T cells was significantly impaired with 8.6- and 3.4-fold reductions in IFNγ and TNF-α, respectively, after TAC-p31 and 61.5- and 15.5-fold reductions in IFNγ and TNF-α, respectively, after TAC (p31+NRPA7) treatment compared to control (**Figure 7I**). Treatment with TAC-p31 alone led to 8.7- and 4-fold reduction in IFNγ and TNF-α, respectively, by NY8.3 T cells further supporting bystander suppression (**Figure 7J**). TAC (p31+NRPA7) treatment led to further attenuation of cytokine secretion by NY8.3 T cells with 266- and 10.3-fold decrease in IFNγ and TNF-α, respectively, compared to control treatment (**Figure 7J**).

To verify that only TACs loaded with cognate MHC-II restricted (p31) epitope can induce Tregs and mediate bystander suppression and not MHC-I restricted (NRPA7) epitope, we utilized the above BDC2.5 and NY8.3 T cell co-adoptive transfer system. Mice were treated with two doses of TAC-HEL, TAC-p31, TAC-NRPA7 after co-transfer of activated p31-specific BDC2.5 CD4 and NRPA7-specific NY8.3 CD8 transgenic T cells into NOD.*scid* mice. Flow cytometric analysis of pancreas on day 9 post-transfer revealed that TAC-p31 treatment led to a 2.5-fold increase in frequency of BDC2.5 Tregs. (**Figure S8A**). However, both TAC-NRPA7 and TAC-HEL failed to induce expansion of BDC2.5 Tregs (**Figure S8A**). IFNγ secretion by BDC2.5 and NY8.3 T cells remained elevated in TAC-NRPA7 treated mice indicating lack of bystander suppression due to the inability to expand antigen-specific Tregs (**Figures S8B, C**). Similar results were seen with TAC-HEL treatment (**Figures S8B, C**). TAC-p31 treatment alone led to 12- and 13.5-fold reduction in IFNγ secretion by BDC2.5 and NY8.3 T cells, respectively, supporting bystander suppression and consistent with previous results (**Figures S8B, C**).

We next evaluated whether the expansion of BDC2.5 Tregs by TAC-p31 treatment was sufficient to prevent transfer of disease mediated by diabetogenic polyclonal T cells from hyperglycemic NOD mice. To this end, activated BDC2.5 T cells were adoptively transferred into NOD.*scid* mice and thereafter treated with three doses of either TAC-HEL or TAC-p31 as depicted in **Figure S9A**. On the day after the last TAC treatment, recipient mice received 10x10^6^ spleen and LN cells from NOD mice that were diabetic (**Figure S9A**). NOD.*scid* recipient mice that were adoptively transferred with the same number of spleen and LN cells from diabetic NOD mice but did not receive prior transfer of activated BDC2.5 T cells, and no TAC-p31 treatment served as controls (**Figure S9A**). Control mice that received only NOD diabetogenic cells developed disease within 62 days of adoptive transfer, whereas TAC-p31 treatment completely protected against transfer of diabetes mediated by polyclonal T cells (**Figure S9B**). TAC-HEL treated mice developed disease within 5 days of NOD diabetogenic cell transfer. The ability of TAC-p31 treatment alone to prevent disease induced by polyclonal NOD diabetogenic T cells further demonstrates strong evidence of bystander suppression.

## Discussion

A major challenge in the development of ASITs is ensuring that antigen is delivered to the right cell type, processed, and presented in a non-inflammatory context to induce tolerance. In this study, we describe TACs as a novel ASIT approach that harnesses the physiological mechanism of RBC clearance to deliver antigens to cognate T cells in a tolerogenic context. Several antigen-coupled apoptotic cell-based approaches have been previously explored to induce antigen-specific tolerance (50). These include conjugation of antigen to leukocytes by chemical methods and covalent or non-covalent linkage of antigen to RBCs (18–20, 23). Chemical methods for antigen conjugation exhibit high variability in coupling efficiency and cause alterations in protein structure and activity (51). The various antigen-coupling methods also increase the risk of inadvertent immune reaction either due to surface exposure of the tethered antigen or dissociation of the antigen leading to uptake by irrelevant cell types. An advantage of our microfluidic squeeze approach to generate TACs is the relative ease in delivering antigens in its native conformation to the RBCs without any treatments to modify the RBC membrane or complex antigen conjugation methods. A notable feature of the cell squeeze platform is that it allows multiplexing several antigens or cargo of diverse types. The interchangeability of payload along with flexibility in choice of target material makes TACs well suited for ASIT therapies for autoimmune diseases involving multiple autoantigens. Furthermore, our approach fully encapsulates the target antigen. Recently, nanoparticles that encapsulate antigen have been used to induce antigen specific tolerance (52–55). However, compared to our approach, the size and route of administration of nanoparticles might lead to non-physiological uptake mechanisms. Additionally, the use of foreign encapsulating materials increases the risk of adverse effects and can lead to tolerability issues in the clinic (56, 57).

We demonstrate that TAC-mediated delivery of autoantigens in a noninflammatory manner led to delay or prevention of onset of T1D induced by activated diabetogenic BDC2.5 CD4 and NY8.3 CD8 T cells, respectively. Induction of tolerance by TAC-p31 was largely dependent on expansion of antigen-specific Tregs and retention of BDC2.5 effectors in the spleen. Unlike published results, CD25 was rapidly downregulated in Tregs after adoptive transfer of activated BDC2.5 T cells in our model irrespective of TAC treatment. Perhaps, differences in stimulation and transfer conditions might account for this discrepancy. While the exact mechanism for splenic effector cell accumulation is unknown, it is very likely mediated by Tregs as they have been shown to potently inhibit pancreatic islet infiltration by suppressing IFNγ production at sites of inflammation and downregulating expression of IFNγ dependent CXCR3 (58). Consistent with the Treg mediated control of pathogenic CD4 T cells, we observed potent inhibition of IFNγ production in the pancreas, reduction in overall numbers of CXCR3 positive effectors and a concomitant decrease in pancreatic islet infiltration in TAC-p31 treated animals.

Notably, TAC-p31 treatment led to an increase in BDC2.5 T-bet^+^ Foxp3^+^ Tregs in the spleen. These T-bet^+^ Tregs also expressed CXCR3 but the overall numbers of “Th1-like” T-bet^+^ Foxp3^+^ CXCR3^+^ Tregs were not significantly increased in the TAC-p31 treated animals. A likely explanation is that this unique subset of T-bet^+^ Tregs migrate to the pancreas in response to CXCR3 chemokines secreted by pancreatic islet cells and APCs. Indeed, several studies have demonstrated preferential enrichment of T-bet^+^ Tregs at sites of Th1-mediated inflammation (48, 59, 60). T-bet^+^ Tregs have enhanced suppressive function and are critical in restraining type 1 inflammation in the pancreas and preventing disease onset in various models of T1D (48, 59). While the suppressive effects of Tregs were not directly examined, we observed increased IL-10 production from splenocytes of TAC-p31 treated mice. Whether the specialized “Th1-like” Tregs accounts for the increased Tregs in pancreas after TAC-p31 treatment needs to be evaluated. Further examination is also needed to determine whether the T-bet^+^ Tregs after TAC-p31 treatment develops into memory Tregs. While BDC2.5 Foxp3^+^ Tregs expressed high levels of co-inhibitory molecules, there was no difference between TAC-p31 and TAC-HEL treatments. A likely explanation is that these markers were upregulated after *in vitro* activation prior to adoptive transfer and tolerance induction did not further enhance their expression. We propose that the major mechanism of tolerance by TAC-p31 is *via* induction of distinct Treg subsets that secrete suppressive cytokines and by increasing the Treg to Teff ratio. However, Foxp3^+^ Tregs numbers and function could wane over time due to loss of CD25 expression and this might account for the delayed onset of T1D in TAC-p31 treated animals.

The success of an ASIT therapy relies on Tregs that can suppress not only cognate autoreactive T cells but also T cells reactive against non-cognate autoantigens *via* bystander suppression. Utilizing the co-adoptive transfer model of BDC2.5 and NY8.3 T cells, we clearly demonstrate bystander suppression by BDC2.5 Tregs after TAC-p31 treatment. The importance of bystander suppression is further supported by the elevated effector BDC2.5 and NY8.3 T cell responses after TAC-NRAP7 treatment alone in the co-adoptive transfer model due to the inability of TAC-NRAP7 to expand antigen-specific Tregs. The strongest evidence for durable BDC2.5 Treg mediated bystander suppression is demonstrated by the ability of TAC-p31 treatment alone to prevent diabetes induced by NOD diabetogenic T cells of different autoreactive specificities. The complete protection against disease induced by transfer of diabetogenic NOD cells was intriguing since TAC-p31 treatment significantly delayed disease but did not prevent T1D development in single BDC2.5 adoptive transfer. One possibility is that the large number of polyclonal diabetogenic T cells transferred provided a bolus of inflammation that served as a source of IL-2, which restored CD25 expression in BDC2.5 Tregs that resulted in further expansion and durable maintenance of these antigen-specific Tregs. While the precise mechanisms of BDC2.5 Treg bystander activity is not known, it is likely mediated by secretion of suppressor cytokines or by competition for IL-2.

Expansion of antigen-specific Tregs by TACs should limit Treg activity to tissue-restricted antigens without inducing global immunosuppression. The ability of TACs to encapsulate both CD4 and CD8 epitopes broadens its translatability for treatment of a broad spectrum of autoimmune disorders that involve reactivity to defined or several autoantigens. A major challenge with the translatability of ASIT therapies to complex autoimmune diseases such as T1D is the lack of knowledge about the driver autoantigen and the heterogenous nature of the disease characterized by presence of multiple autoantigens (61). For diseases such as T1D that is defined by distinct stages (62, 63), TACs have the potential to induce tolerance in recent onset patients. In newly diagnosed patients characterized by distinct biomarkers, incorporation of multiple autoantigens that confer bystander tolerance can limit epitope spreading and delay progression of disease or preserve beta cell function. Furthermore, the flexibility in payload allows incorporation of pharmacologic agents or immunomodulators that can enhance tolerance and confers an advantage for TACs over existing ASIT therapies.

Our study demonstrates the potential of TACs as a safe, and modular platform where antigens can be easily swapped out to provide tailored therapies for a wide range of autoimmune disorders.

## Data Availability Statement

The original contributions presented in the study are included in the article/**Supplementary Material**. Further inquiries can be directed to the corresponding authors.

## Ethics Statement

The animal studies were carried out according to protocols established by the American Association for Laboratory Animal Science and reviewed and approved by the Institutional Animal Care and Use of Laboratory Animal Committee (IACUC) at SQZ Biotechnologies.

## Author Contributions

Conceptualization: SJ. Formal Analysis: SJ, CR, JDC, GS, JNC, and PC. Funding Acquisition: AS and HB. Investigation: CR, JDC, GS, JNC, PC, HL, and DK. Methodology: SJ. Project Administration: SJ and HB. Supervision: SJ. Validation: SJ. Visualization: CR. Writing – original draft: SJ and CR. Writing – reviewing and editing: CR, HB, and AS. All authors contributed to the article and approved the submitted version.

## Funding

The authors declare that this study received funding from the Juvenile Diabetes Research Foundation (JDRF). The funder was not involved in the study design, collection analysis, interpretation of data, the writing of this article or the decision to submit it for publication.

## Conflict of Interest

All authors were employed by SQZ Biotechnologies Company.

## Publisher’s Note

All claims expressed in this article are solely those of the authors and do not necessarily represent those of their affiliated organizations, or those of the publisher, the editors and the reviewers. Any product that may be evaluated in this article, or claim that may be made by its manufacturer, is not guaranteed or endorsed by the publisher.
