# Supplementary material for: Engineered RBCs Encapsulating Antigen Induce Multi-Modal Antigen-Specific Tolerance and Protect Against Type 1 Diabetes
Source: Front Immunol. 2022 Apr 4;13:869669. doi: 10.3389/fimmu.2022.869669 (PMC9014265; doi:10.3389/fimmu.2022.869669)
Supplement: Supplementary file 1 [file DataSheet_1.docx]

# **Supplementary Figures**

**Figure S1. Annexin V and OVA-647 delivery in SSC^lo^ or SSC^hi^ cell populations in unprocessed and squeezed RBCs** Representative flow cytometry plots showing Annexin V and OVA-647 positive cells in SSC ^hi^ and SSC ^lo^ cell populations in unprocessed and squeezed RBCs (**A**). Percentage of Annexin V positive cells (**B**) and OVA-647 delivery (**C**) in unprocessed RBCs and squeezed RBCs gated on SSC^hi^ or SSC^lo^ cells.


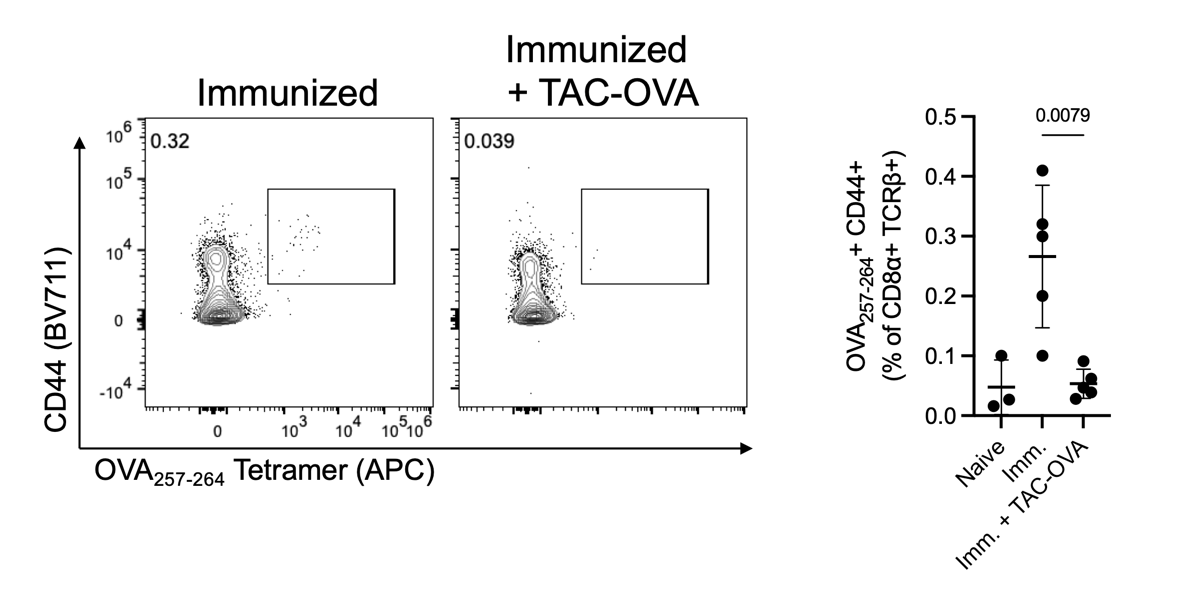
s

**Figure S2. TAC-OVA treatment reduces the frequency of OVA-specific CD8+ T cells in the draining LN.** C57BL6/J mice were treated as described in **Fig. 2A**. Draining LN were collected, and lymphocytes were analyzed by tetramer staining with MHC-I immunodominant epitope of Ovalbumin. Cells were pre-gated on live CD3^+^ TCRβ^+^ CD8α^+^ CD4^-^. Statistics calculated by Mann-Whitney U test. Data is representative of at least 3 independent experiments. Error bars represent mean+/- S.D.

**Figure S3. TAC-OVA suppresses antigen-specific T cell responses in the spleen.** (**A-B**) C57BL/6 mice were either untreated or treated on days -7 and -4 with TAC-OVA and either immunized subcutaneously on day 0 with OVA/CFA or left unimmunized. 7d later, OVA-specific T cell responses were evaluated in spleen by ELISpot. IL-2 (**A**) and IFNγ (**B**) responses in spleen after *in vitro* restimulation with OVA protein (**A**) or SIINFEKL peptide (**B**). n=3 for naïve and n=5 for other groups. Statistics calculated by Mann-Whitney U test between immunized and immunized + TAC-OVA groups. Data are representative of at least two independent experiments. Error bars represent mean +/- S.D.

**Figure S4. TACs loaded with OVA induce potent suppression of antigen-specific immune responses compared to soluble OVA treatment.** C57BL6/J mice were immunized with OVA emulsified in CFA as described in Fig. 2. On days -7, -4, and -1 relative to immunization (day 0), mice were treated with 100μg of OVA protein or approximately 100μg of OVA encapsulated in 1x10^9^ TAC-OVA (squeezed at 50uM with OVA) by *i.v.* injection. 7 days after immunization, IL-2 (**A**) and IFNγ (**B**) producing cells were assayed by ELISpot after restimulation with OVA protein (**A**) or SIINFEKL peptide (**B**). Statistics calculated by one-way ANOVA with Tukey’s Multiple Comparisons test. n=5-6 mice per group.

**
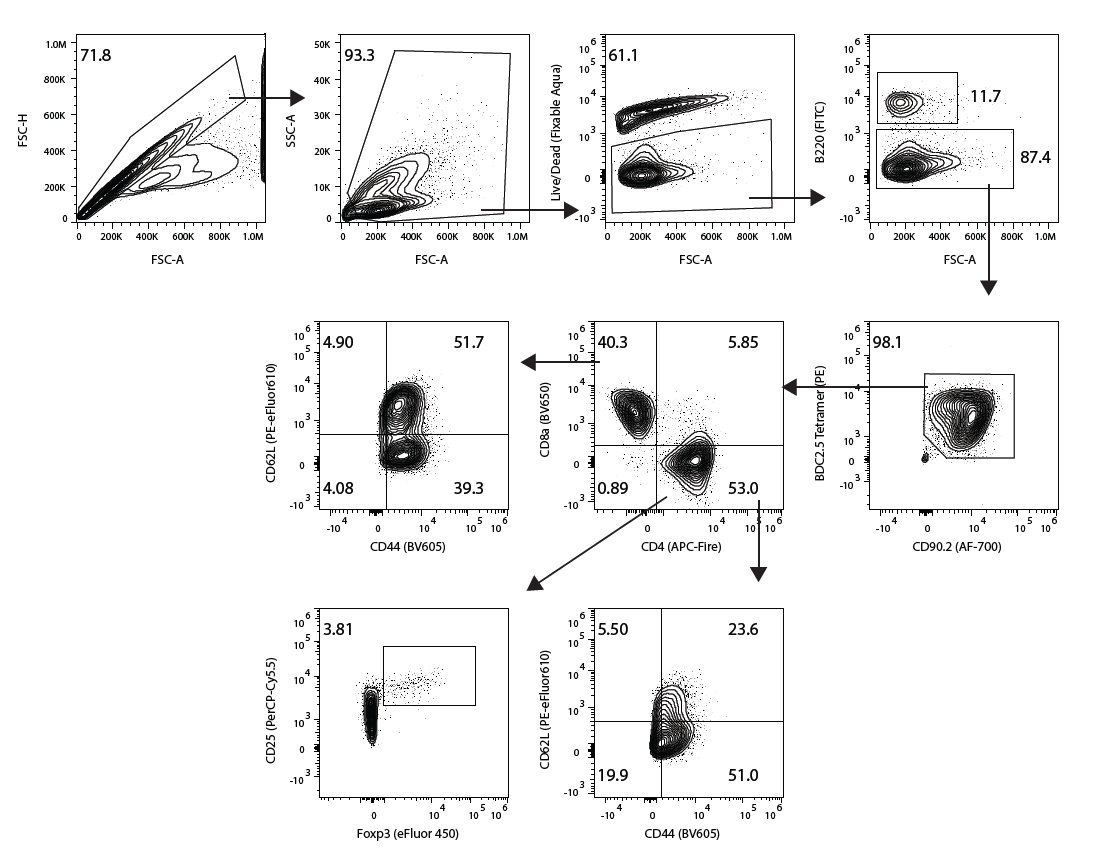
**

**Figure S5. Phenotypic characterization of *in vitro* stimulated BDC2.5 T cells.** Lymphocytes were isolated from spleen and various lymph nodes of BDC2.5 transgenic mice and cultured with p31 peptide mimetope for 4 days. The purity and activation status of BDC2.5 T cells were analyzed by flow cytometry prior to adoptive transfer into NOD. *scid* recipients.

**
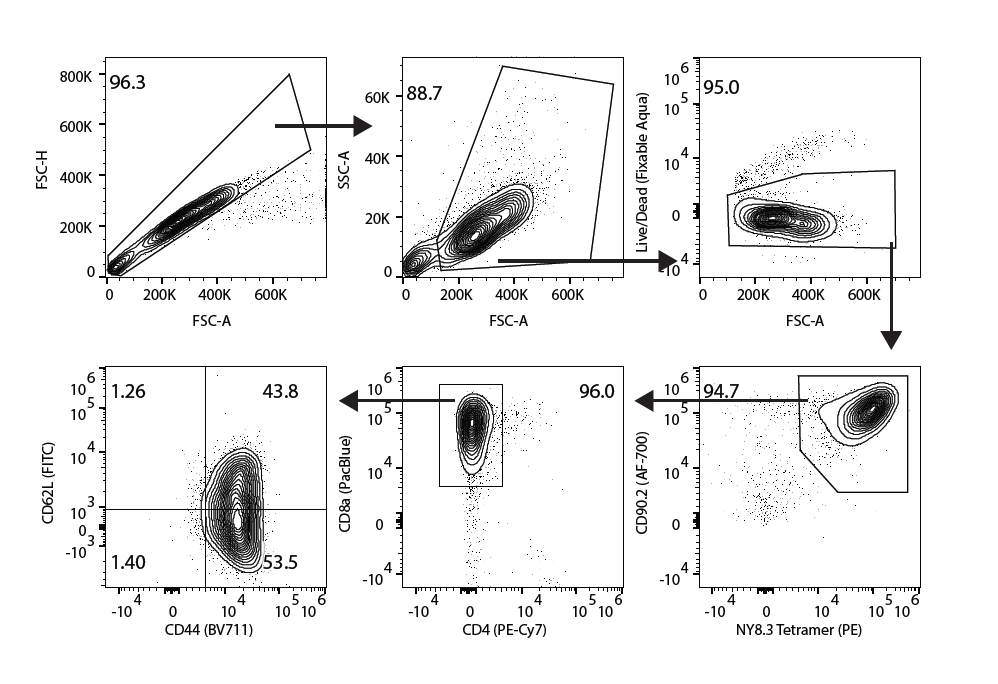
**

**Figure S6. Phenotypic characterization of *in vitro* stimulated NY8.3 T cells.** Lymphocytes were isolated from spleen and various lymph nodes of BDC2.5 transgenic mice and cultured with NRPA7 peptide mimetope for 4 days. Dead cells were removed from culture, and the purity and activation status of NY8.3 CD8 T cells were analyzed by flow cytometry prior to adoptive transfer into NOD. *scid* recipients.

**
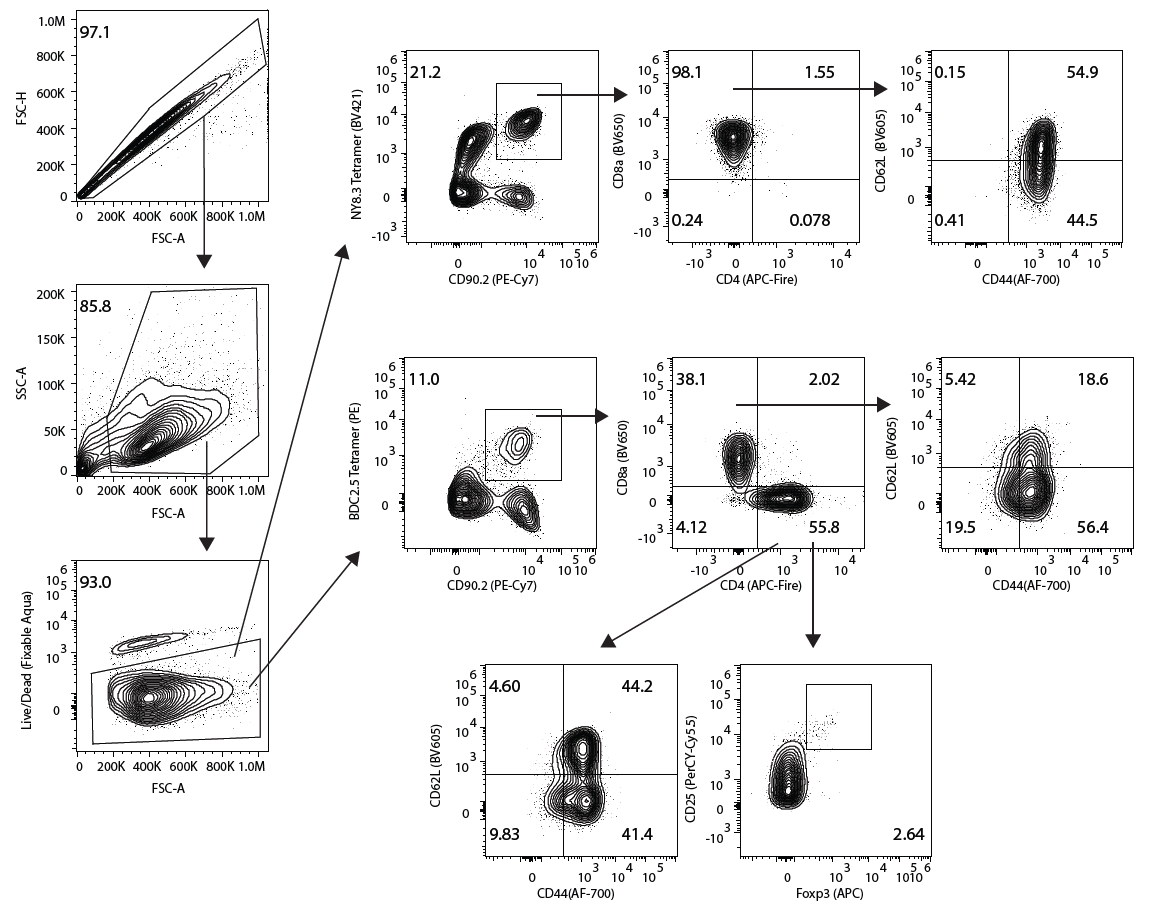
**

**Figure S7. Phenotypic analysis of activated BDC2.5 and NY8.3 used for co-transfer.** BDC2.5 and NY8.3 cells were isolated from respective transgenic mice and cultured separately for 4 days with p31 or NY8.3 mimetope peptide, respectively. Dead cells were removed from the NY8.3 T cell culture. Thereafter BDC2.5 and NY8.3 cell cultures were mixed in equal ratios and analyzed by flow cytometry prior to adoptive transfer into NOD.*scid* recipients.

**Figure S8. TAC loaded with MHC-I restricted NRPA7 epitope did not exhibit bystander suppression.** 5x10^6^ each activated BDC2.5 and NY8.3 T cells were co-transferred into NOD.*scid* mice and treated with TAC-HEL (control), TAC-p31, or TAC-NRPA7 within two hours and on day 2. T cell phenotype was assessed in the pancreas on day 9 post-transfer. (**A**) Foxp3^+^ Tregs among CD44^hi^ BDC2.5 cells (CD90.2^+^ CD4^+^ BDC2.5 Tetramer^+^ CD44^hi^) among the indicated treatment groups. Proinflammatory cytokine secretion by (**B**) BDC2.5 and (**C**) NY8.3 cells after stimulation with (**B**) p31 or (**C**) NRPA7 peptide by cells from the indicated treatment groups. n=2 pools of cells from 4 animals each; total n=8.


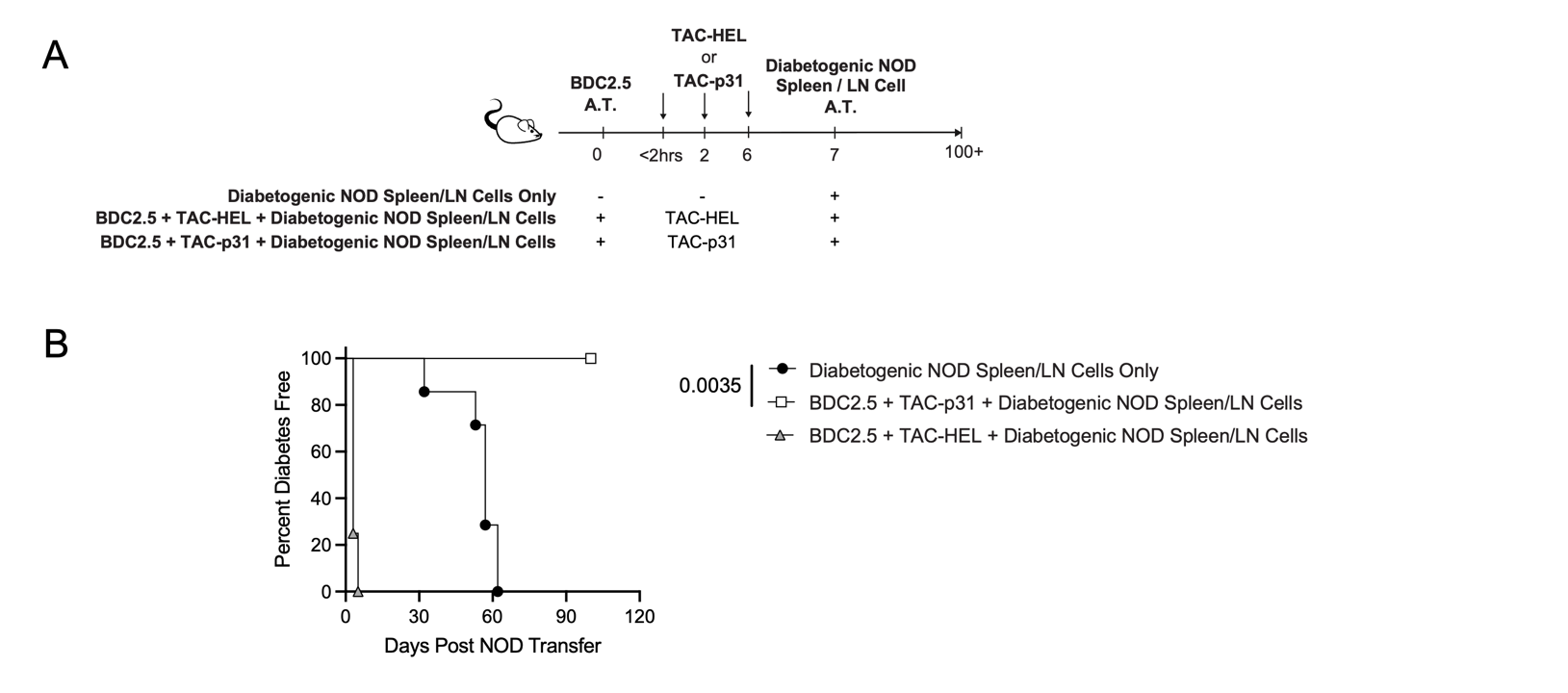


**Figure S9. TAC-p31 treatment prevents transfer of diabetes by polyclonal pathogenic T cells. (A)** On day 0, NOD.*scid* mice were adoptively transferred with 5x10^6^ activated BDC2.5 cells and then treated with either 1x10^9^ TAC-p31 or TAC-HEL within 2 hours. Mice were given additional TAC doses on days 2 and 6. On the day following TAC treatment, 10x10^6^ spleen and LN cells from diabetic NOD mice (BDC2.5 + TAC-p31 + diabetogenic NOD spleen + LN cells or BDC2.5 +TAC-HEL + diabetogenic NOD spleen + LN cells) were adoptively transferred into the recipients by tail vein injection. NOD.scid recipient mice that received only diabetogenic NOD cells (diabetogenic NOD spleen + LN cells only) without prior BDC2.5 adoptive transfer or TAC treatment served as controls (**B**) Diabetes incidence was monitored for 100 days following challenge with NOD diabetogenic cells in the various cohorts of mice as described in A. Log-rank (Mantel-Cox) test. NOD transfer only n=7 ; BDC2.5 + TAC-p31 + NOD n=4 ; BDC2.5 + TAC-HEL + NOD n=8.

# **Supplementary Tables**

**Table S1**

| **Treatment** | **N** | **Perivascular/ periductular, infiltration, mononuclear cell** | **Islet of Langerhans, infiltration, mononuclear cell** |
| --- | --- | --- | --- |
| TAC-HEL | 2 | 2 | 2 |
|  |  | 4 | 4 |
| TAC-p31 | 2 | 1 | 2 |
|  |  | 1 | 1 |
| 1=Minimal; 2=Mild; 3=Moderate; 4=Marked | | |  |
|  | | |  |

Severity of insulitis in TAC-HEL and TAC-p31 treated mice.

**Table S2**

| **Treatment** | **N** | **Perivascular/ periductular, infiltration, mononuclear cell** | **Islet of Langerhans, infiltration, mononuclear cell** |  |  |
| --- | --- | --- | --- | --- | --- |
| Empty TAC | 2 | 2 | 4 |  |  |
|  |  | 2 | ### |  |  |
| TAC-NRPA7 | 2 | 1 | 2 |  |  |
|  |  | 0 | 1 |  |  |
|  |  |  |  |  |  |
| 1=Minimal; 2=Mild; 3=Moderate; 4=Marked | | |  |  |  |
| ### = Islets were not confidently identified due to high levels of mononuclear cell infiltration | | | | | |
|  | | | | | |
|  | | | | | |

Severity of insulitis in TAC-NRPA7 treated mice and control (Empty TAC).

**Table S3**

Antibodies and tetramers used for flow cytometry.

| **Target** | **Fluorophore** | **Clone** | **Manufacturer** | **Cat No.** | **Dilution** |
| --- | --- | --- | --- | --- | --- |
| BDC2.5 Tetramer | PE | na | MBL | TS-M727-1 | 1:10 |
| NY8.3 Tetramer | PE | na | MBL | TB-M553-1 | 1:20 |
| NY8.3 Tetramer | APC | na | MBL | TB-M553-2 | 1:20 |
| NY8.3 Tetramer | BV421 | na | MBL | TB-M553-4 | 1:20 |
| O­­VA_257-264_ Tetramer | APC | na | MBL | TB-5001-2 | 1:10 |
| B220 | FITC | RA3-6B2 | Biolegend | 103206 | 1:200 |
| CD103 | PE-eFluor610 | 2E7 | eBioscience | 61-1031-82 | 1:200 |
| CD11b | PE-Cy7 | M1/70 | Biolegend | 101215 | 1:200 |
| CD11b | PerCP-Cy5.5 | M1/70 | Biolegend | 101228 | 1:200 |
| CD11c | BV510 | N418 | Biolegend | 117338 | 1:200 |
| CD11c | BV650 | N418 | Biolegend | 117339 | 1:200 |
| CD11c | PerCP-Cy5.5 | N418 | Biolegend | 117328 | 1:200 |
| CD138 | APC-Cy7 | ZET | Biolegend | 148224 | 1:200 |
| CD146 | PE-Cy7 | ME-941 | Biolegend | 134714 | 1:200 |
| CD19 | FITC | ID31CD19 | Biolegend | 152404 | 1:200 |
| CD25 | PerCP-Cy5.5 | PC61 | Biolegend | 102030 | 1:200 |
| CD3 | PE-eFluor610 | 145-2C11 | eBioscience | 61-0031-82 | 1:200 |
| CD3 | PE-Cy7 | 17A2 | Biolegend | 100220 | 1:200 |
| CD31 | APC-Cy7 | 390 | Biolegend | 102440 | 1:200 |
| CD39 | PE-Cy7 | Duha59 | Biolegend | 143806 | 1:200 |
| CD4 | BV605 | GK1.5 | Biolegend | 100451 | 1:200 |
| CD4 | BV711 | GK1.5 | Biolegend | 10047 | 1:200 |
| CD4 | PE-Cy7 | RM4-5 | Biolegend | 100528 | 1:200 |
| CD4 | APC-Fire | GK1.5 | Biolegend | 100460 | 1:200 |
| CD4 | PerCP-Cy5.5 | GK1.5 | Biolegend | 100434 | 1:200 |
| CD44 | BV711 | IM7 | Biolegend | 103057 | 1:200 |
| CD44 | BV605 | IM7 | Biolegend | 103047 | 1:200 |
| CD44 | AF700 | IM7 | Biolegend | 103026 | 1:200 |
| CD45 | BV650 | 30-f11 | Biolegend | 103151 | 1:200 |
| CD45 | AF700 | 30f-11 | Biolegend | 103128 | 1:200 |
| CD47 | BV421 | miap301 | Biolegend | 127527 | 1:10 |
| CD62L | FITC | MEL-14 | Biolegend | 104406 | 1:200 |
| CD62L | PE-eFluor610 | MEL-14 | eBioscience | 61-0621-82 | 1:200 |
| CD62L | BV605 | MEL-14 | Biolegend | 104438 | 1:200 |
| CD68 | AF488 | FA-11 | Biolegend | 137012 | 1:200 |
| CD8a | PE-eFluor610 | 53-6.7 | eBioscience | 61-0081-82 | 1:200 |
| CD8a | PacBlue | 53-6.7 | Biolegend | 100725 | 1:200 |
| CD8a | BV605 | 53-6.7 | Biolegend | 100784 | 1:200 |
| CD8a | BV650 | 53-6.7 | Biolegend | 100742 | 1:200 |
| CXCR3 | APC | CXCR3-173 | Biolegend | 126512 | 1:200 |
| CD90.2 | AF700 | H1.2F3 | Biolegend | 140324 | 1:200 |
| CD90.2 | PE-Cy7 | 53-2.1 | eBioscience | 25-0902-82 | 1:200 |
| CTLA4 | PE-eFluor610 | UC10-4B9 | eBioscience | 61-1522-82 | 1:200 |
| F4/80 | APC | BM8 | Biolegend | 123116 | 1:200 |
| Foxp3 | eFluor450 | fjk-16s | eBioscience | 48-5773-82 | 1:200 |
| Foxp3 | APC | FJK-16s | eBioscience | 17-5773-82 | 1:200 |
| GITR | BV711 | DTA-1 | BD | 563390 | 1:200 |
| IA/IE | BV605 | M5/114.15.2 | Biolegend | 107632 | 1:200 |
| IA/IE | BV510 | M5/114.15.2 | Biolegend | 107636 | 1:200 |
| IFNγ | PE-eFluor610 | XMG1.2 | eBioscience | 61-7311-82 | 1:200 |
| LY6G | APC-Cy7 | 1A8 | Biolegend | 127623 | 1:200 |
| Rat IgG2a Isotype | BV421 | RTK2758 | Biolegend | 400536 | 1:10 |
| SIRPα | AF700 | P84 | Biolegend | 144021 | 1:200 |
| Tbet | FITC | 4B10 | Biolegend | 644812 | 1:200 |
| TCRβ | AF700 | H57-597 | Biolegend | 109224 | 1:200 |
| TNF-α | PE-Cy7 | MP6-XT22 | Biolegend | 506324 | 1:200 |
| XCR1 | APC-Cy7 | ZET | Biolegend | 148224 | 1:200 |
